# Towards implementing new payment models for the reimbursement of high-cost, curative therapies in Europe: insights from semi-structured interviews

**DOI:** 10.3389/fphar.2024.1397531

**Published:** 2025-01-20

**Authors:** Thomas Desmet, Sissel Michelsen, Elena Van den Brande, Walter Van Dyck, Steven Simoens, Isabelle Huys

**Affiliations:** ^1^ Clinical Pharmacology and Pharmacotherapy, Department of Pharmaceutical and Pharmacological Sciences, KU Leuven, Leuven, Belgium; ^2^ Healthcare Management Centre, Vlerick Business School, Ghent, Belgium

**Keywords:** semi-structured interviews, managed entry agreements (MEA), annuity, spread payment, outcome-based reimbursement, advanced therapy medicinal product (ATMP), curative therapy

## Abstract

**Background:**

New ways of reimbursement for high-cost, one-shot curative therapies such as advanced therapy medicinal products (ATMPs) are a growing area of interest to stakeholders in market access such as industry representatives, legislative and accounting experts, physicians, hospital managers, hospital pharmacists, patient representatives, policymakers, and sickness funds. Due to the complex nature of ATMPs, new payment models and reimbursement modalities are proposed yet not widely applied across Europe.

**Objectives:**

This study aimed to elicit opinions on and insights into the governance aspect of implementing outcome-based spread payments (OBSP) in Belgium for the reimbursement of innovative therapies. Stakeholders’ responsibilities and roles were analysed and proposed solutions or general beliefs were assessed to identify necessary or sufficient conditions to establish outcome-based spread payments.

**Methods:**

Semi-structured interviews (n = 33) were conducted with physicians (n = 2), hospital pharmacists (n = 4), hospital managers (n = 2), Belgian policymakers (n = 6), legislative experts (n = 2), accounting experts (n = 5), representatives of patients (n = 3), of industry (n = 5), and sickness funds (n = 4). The interviews took place between July 2020 and October 2020. The framework method analysis was performed using Nvivo software (version 20.4.1.851). Statements were allocated into six main topics: payment structure, spread payments, outcome-based agreements, governance, transparency, and regulation.

**Results:**

Interviews revealed the necessary conditions that, fulfilled together, are seen to be sufficient for the successful implementation of OBSP, including consensus on pricing, payment logistics, robust data infrastructure and financing, clear agreement terms (duration, outcome parameters, payment triggers), long-term patient follow-up solutions, an external multi-stakeholder governance body, and transparency regarding agreement types.

**Conclusion:**

Despite the interest, the effective implementation of OBSP falls behind due to a lack of consensus on how this new reimbursement method can be a sustainable solution. By stating the necessary conditions that, when fulfilled together, are deemed sufficient for successful OBSP implementation, this study provides a framework towards overcoming implementation barriers and realizing the potential of OBSP in transforming healthcare reimbursement practices.

## 1 Introduction

To date, 26 advanced therapy medicinal products (ATMPs) have been approved (European Medicines Agency, 2024) and many more are expected to enter the stage of regulatory approval in Europe in the coming years. In general, patient access may occur prior to market authorisation via participation in clinical trials, via dedicated national early access programs, or after market authorisation via regular or fast access programs. However, standard reimbursement systems face many challenges when dealing with reimbursement applications for potential one-time high-cost therapies like ATMPs. The remaining clinical uncertainties associated with these novel therapies prior to or even at the time of regulatory approval and the often high upfront cost have led to managed entry agreements (MEAs) being the standard tool in various countries to enable access whilst addressing these uncertainties on a contractual basis and managing budget impact (Gerkens et al., 2017). The use of MEAs was intended to be an exception; however, their widespread application is placing pressure on health systems as public funding is used to finance and administratively follow up on these confidential contracts. This has inspired the development of alternative payment structures, next to the pure financial-based agreements such as payer reinsurance, pooling budgets, spread (annuity-based) payments, and outcome-based agreements (OBAs) to mitigate the unaffordability of high-cost, one-time, and possibly curative therapies (Precision Financing Solutions for Durable, 2019; Carr and Bradshaw, 2016; Marsden et al., 2016; Kefalas et al., 2018; Jorgensen et al., 2019; AMCP Partnership Forum, 2019; Annemans and Pani, 2017).

Enthusiasm for novel payment structures is high (Picecchi et al., 2020; Ronco et al., 2021; Callenbach et al., 2024). Still, scepticism also rises due to several barriers to implementing them in the current healthcare system (Barlow et al., 2019; Hanna et al., 2018; Carlson et al., 2017). These barriers include the need for additional data collection and required infrastructure, high administrative burden and cost, the influence of newly approved therapies on agreement terms, governance procedures of negotiating and maintaining agreements, the necessary legislative changes, compliance with current national and European accounting rules and determining financial terms of contracts while considering budget cycle timelines (Gerkens et al., 2017; AMCP Partnership Forum, 2019; Garrison et al., 2013; Jönsson et al., 2019; Adamski et al., 2010). This trend is also visible in the existing literature where several authors propose novel payment structures for gene therapies. However, information is still lacking on their practical implementation within healthcare systems (Carr and Bradshaw, 2016; Marsden et al., 2016; Hettle et al., 2017; Edlin et al., 2014; Sachs et al., 2018; Faulkner et al., 2016) as described in a previously published systematic literature review (Michelsen et al., 2020).

Paying in instalments and making it dependent on real-world treatment outcomes is one solution, called outcome-based spread payments (OBSPs) (Precision Financing Solutions for Durable, 2019; Carr and Bradshaw, 2016; Marsden et al., 2016; Kefalas et al., 2018; Edlin et al., 2014; Jorgensen and Kefalas, 2017). This approach is a risk-sharing structure, that can be concluded in an agreement where the payer will only pay the company for the treatment if certain outcome criteria are met that support the effectiveness of the treatment. These agreements can be implemented through annuity-style payments where payments are performed yearly (Garrison et al., 2013; Hettle et al., 2017; Edlin et al., 2014). The primary objective of this study was to explore how these new payment modalities, like OBSPs, can be practically implemented in the Belgian social security healthcare system, including the legislative and organisational feasibility of such payment processes. Therefore, stakeholders were questioned about the needed infrastructure and governance systems for data collection and needed changes in the organisation (monetary streams, financing, and management). Second, stakeholders’ responsibilities and roles were analysed to refine the required terms between the different stakeholders to allow for spread payments. Proposed solutions or general beliefs were assessed to determine whether they are necessary or sufficient to establish OBSPs. Although the interview focus was on Belgian stakeholders, these results may also be applicable in other countries with similar healthcare systems.

## 2 Methods

### 2.1 General research design and data collection

This study employed a mixed-method approach, commencing with a previously published systematic literature review (Michelsen et al., 2020) followed by semi-structured interviews. Approval from the ethics committee was obtained on the 14th of July 2020 (S64168). The interview guide, which allowed for some open discussion with the representatives from every stakeholder group, was based on the findings from the literature review and round table discussions (Maes et al., 2019). The interview guide comprises a set of standardized open-ended questions, along with more nuanced questions tailored for each stakeholder group. The topics of the interview guide can be found in the Appendix.

### 2.2 Interviewee selection and recruitment

**FIGURE 1:**
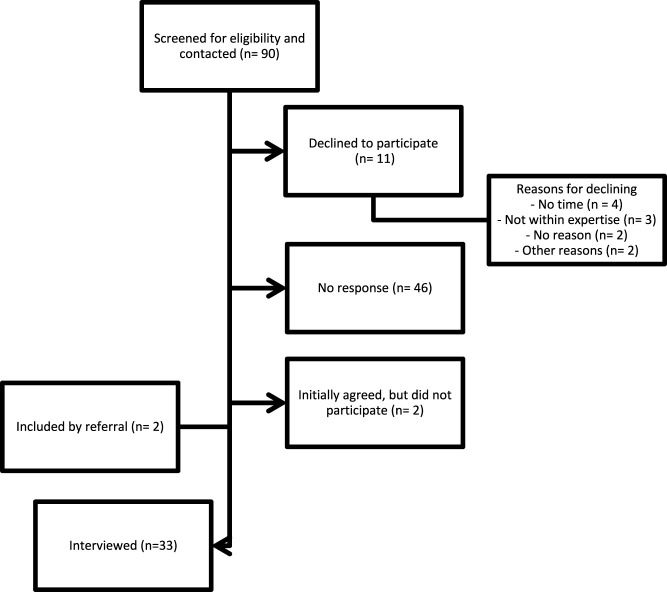
A flow diagram of the participants’ recruitment for the semi-structured interviews.

Figure 1, participants were identified and selected via the **purposeful sampling technique**, a widely used method in qualitative research for the identification and selection of information-rich cases for the most effective source of limited cases. Specifically, **heterogeneous sampling or maximum variation sampling** was applied in this study to capture a wide range of perspectives from especially knowledgeable stakeholder groups related to the subject of the study (Palinkas et al., 2015). **Inclusion** criteria entailed being directly or indirectly involved in ATMP development, dispensing, use, policy- and/or decision-making. Stakeholders were **excluded** if they were unfamiliar with the European situation, had insufficient experience or knowledge concerning ATMPs or had insufficient knowledge of English or Dutch. A predefined sample of approximately 30 stakeholders, with every stakeholder group represented to ensure a **representative sample** was aimed for until data saturation was reached as described in supplementary material ‘Sample size’. Participants could also be contacted **based on referrals** by others who have already been interviewed. The study team extended invitation letters to potential participants through email. Contact information was sourced from publicly available channels or the authors’ network connections. Afterwards, the possible participants were sent an email with the information letter, informed consent form, and interview guide in their preferred language (Dutch or English). This email explicitly stated that the participants could ask any questions they might still have regarding the study.

Those stakeholders were physicians from different therapeutic domains, hospital pharmacists, hospital managers from different Belgian hospitals, domain-specific legislative experts with expertise in reimbursement of medicines (i.e., academia) and/or the accounting of expenditures of medicines in the European Union (i.e., European Commission, academia), domain-specific accounting experts, representatives of umbrella industry associations (Pharma.be and the European Federation of Pharmaceutical Industries and Associations (EFPIA)), as well as those involved in market access on a management level of individual pharmaceutical companies, hereafter called manufacturers, developing innovative medicines that may be subject to OBAs, representatives of different patient organisations from different therapeutic areas where gene therapies are available or will be available in the near future, representatives of governmental organisations involved in policy-making regarding ATMPs: National Institute for Health and Disability Insurance (NIHDI), Belgian Healthcare Knowledge Centre (KCE), the Federal Public Service, and of healthdata. be (part of Sciensano), and representatives of different sickness funds involved in the decision-making of ATMPs.

### 2.3 Conduct of the interviews

The interviews took place between July 2020 and October 2020. Skype/telephone interviews were conducted during this time as the federal measures and restrictions for the COVID-19 pandemic applied. The duration of the interviews was approximately 1 hour. Two pilot interviews conducted in advance were included in the analysis as no changes to the interview guide were deemed necessary.

### 2.4 Data analysis

Following the interviews, the audio recordings were transcribed ad verbatim. Transcripts were pseudonymized, all names and references to participants were removed and every participant received an identifier. Next, the transcripts were analysed using framework analysis (Gale et al., 2013), a qualitative content analysis method, using Nvivo software (version 20.4.1.851). Complementary deductive codes, drafted based on the interview guide, and inductive codes (themes), arising throughout the process of analysing the transcripts, were used to index the raw data. Afterwards, codes were grouped into categories of correlated topics and concepts and charted into a matrix. This resulted in a total of six topics: payment structure, spread payments, OBAs, governance, transparency, and regulation.

## 3 Results

A total of 32 semi-structured interviews with 33 interviewees were conducted in English or Dutch with different European stakeholders with relevance to the Belgian context (five industry representatives, two legislative and five accounting experts), as well as Belgian stakeholders (two physicians, two hospital managers, four hospital pharmacists, three patient representatives, six policymakers, and four sickness funds representatives) until theoretical saturation was reached (Kerr et al., 2010). Statements included in the article are selected to highlight key perspectives, while the section as a whole explores a broader range of views on the topic.

### 3.1 The role of hospitals in the payment flow

**FIGURE 2:**
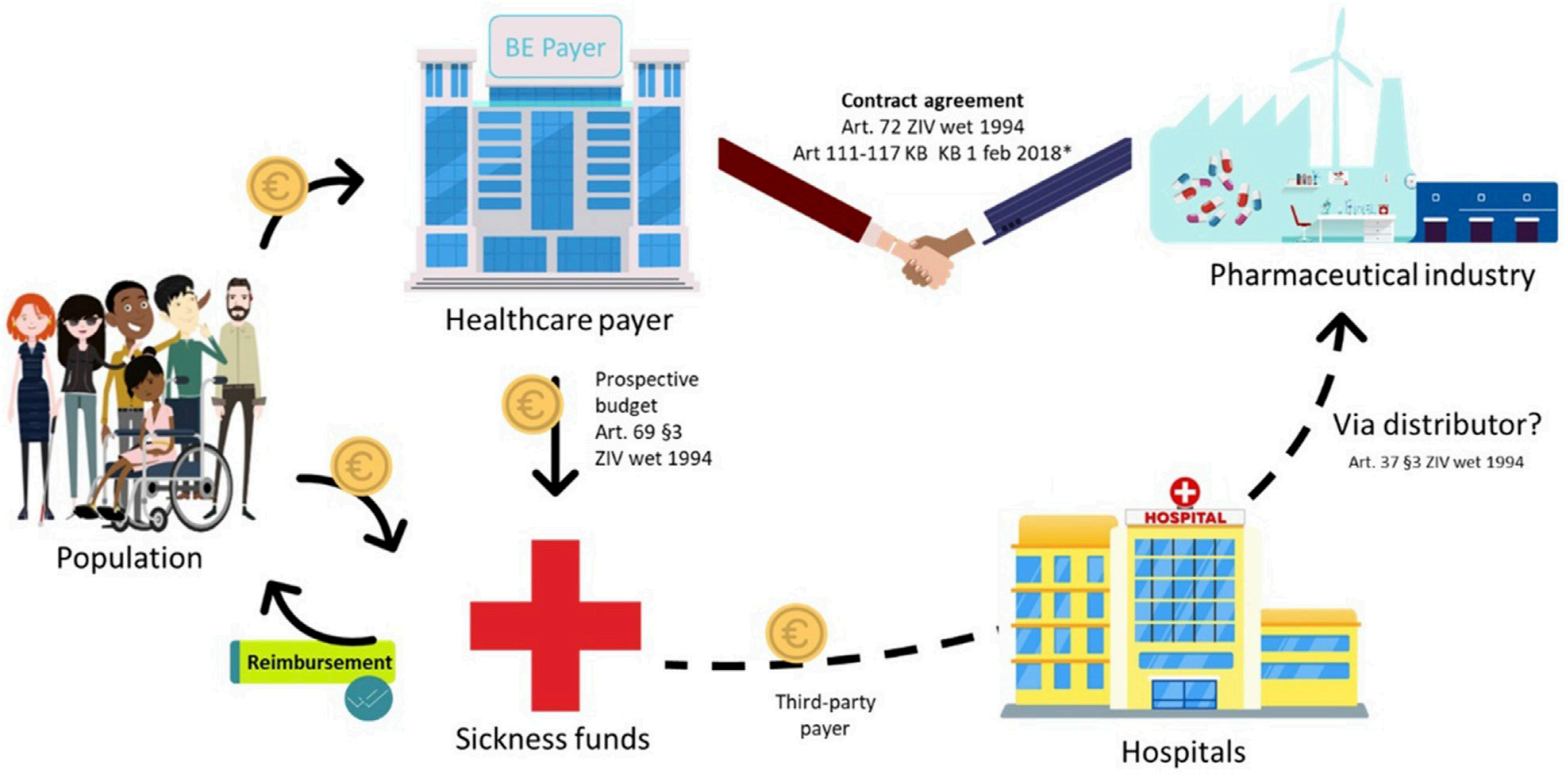
Standard payment process for class A, hospital-administered ATMPs in Belgium. NIH: National Institute for Health; ATC: Anatomical Therapeutic Chemical; ZIV: Sickness Insurance *‘special, “new” and “expensive” pharmaceuticals can be billed to the government per used product–list with ATC codes in Appendix IV of KB 1 February 2018 (1,2) - The hospital bills the sickness funds once per month (3) Note: this figure is based on (1) Forfaitarisering van de farmaceutische specialiteiten in het ziekenhuis (RIZIV); (2) KCE Report 302A; (3) Instructies voor de facturatie op magnetische of elektronische drager (RIZIV).

The current Belgian healthcare system is characterised by a third-party payer system for ATMPs: the hospital procures such therapies from manufacturers, and reimbursement is facilitated through the sickness funds (the third-party payers), which get their budget from the public social security institution NIHDI. This system involves advance payments and settlements in the same calendar year, which poses a first challenge for spread payments. Interviewees identified a second issue, specifically concerning the national payer’s lack of direct access to data on using ATMPs. Therefore, the payer must rely on sickness funds to provide such data for refund calculations, causing delays in determining budgets for those same sickness funds. This exemplifies the scepticism expressed by some interviewees regarding the added value of the sickness funds in this current payment system (Figure 2).

A third challenge lies in the belief that hospital systems are not prepared for administrative management of spread payments, especially not if several medicines have their unique payment terms. To cover expensive, one-time drugs it was said that hospitals should be able to pay in instalments while they cannot be expected to prefinance such therapies fully. It was also pointed out that such therapies could be processed as part of the Budget Financial Resources, enabling hospitals to receive a lump sum allowance to cover such drug expenses.

*“Hospitals are not the government’s bank.”*

*“The hospital will not take any financial risks.”*

Additionally, interviewees raised concerns about hospitals’ role in the financing process and suggested that hospitals be removed from the payment flow altogether (Figure 2).

However, excluding hospitals from the payment flow does not necessarily ensure a reduction in the administrative burden. While recognizing the need for hospital financing reform, a hospital pharmacist emphasized that completely bypassing hospitals might be impractical. Today, hospitals secure discounts through their established discount agreements via their hospital pharmacy, which accounts for 16% of hospitals’ income.

### 3.2 Spread payments

The interviews revealed a lack of consensus on the financial terms and the goal of spread payments. Spread payments have been presented as an incentive to collect data, however, some stakeholders feel they should tackle purely budgetary problems and provide risk mitigation.

Although spread payments are not seen as the solution to reduce drug prices, industry representatives see it as a way to ensure affordability and smooth out peak budget impact for the payer, while also creating predictable revenue streams for manufacturers. They also caution that it should not become a systematic solution for any ATMP.

*“There will always be tension between what healthcare system considers appropriate as price level and what a company wants as a return on their investments.”*

A second group views spread payments as an intermediary solution to make treatment available as they see it as a stay of execution (payment of a debt). However, it is important to note that the total cumulative cost could stay the same or increase since the manufacturers may be exposed to higher risk or have to fund the therapy themselves (incurring the cost of credit). Some worry that spread payments could burden any future government because they see it as a deferral of payment which limits the budget for subsequent years and the freedom to operate of the next minister. Combining spread payment with an outcome-based mechanism could potentially soften this burden, as often suggested by interviewees.

A third group does not favour spread payments because they believe that manufacturers will always try to maximize their profits, and any price increase will be hidden by having the spread payment in place.

*“It is a facade to carry out price increases.”*

It is crucial to consider whether the additional management costs of staggering payments over time outweigh the advantages of not having to free up the entire budget immediately. Some interviewees argued that the first payment should be a small upfront payment to make treatment available since effectiveness has not yet been proven. Others preferred a bigger lump-sum payment at the start given some risk the government must take, with follow-up reimbursements depending on effectiveness. Some interviewees considered this to be the wrong way of doing it. They emphasized that from a payer’s perspective only what is supported by evidence should be paid for, and payments can increase as the evidence improves.

#### 3.2.1 Duration and intervals of spread payments

The interviews revealed two possible intervals for spread payments: yearly payments or on a case-by-case basis, i.e., in line with consultations. According to the interviews, the optimal duration for spread payments is between two and 10 years. Both extremes have pros and cons, and a balance must be found to control the risks both at the corporate and government levels. The optimal duration depends on factors like the population, the disease, the survival rate, the outcome to be measured, the likelihood that patients will attend follow-up visits, the ageing of the drug, market competition, as well as the opportunity cost and inflation.

As one interviewee summarized, there is probably a limited timeframe, minimum and maximum duration, for annuity payments to make any sense. Making commitments too far into the future should be avoided since the circumstances might change. For instance, a patient might move abroad, which poses two problems: one in follow-up in case of an OBA and one in payment responsibility in case of a spread payment. There is consensus that data collection during the follow-up period should occur in the country of residence without specific reference to insurance. Some believed that the country where the patient is insured is responsible for the therapy payments, citing the cross-border healthcare directive, while others referred to the country of therapy administration.

### 3.3 Outcome-based managed-entry agreements

Interviewees pointed out that thanks to MEAs, therapies can reach patients when manufacturers may convince payers that the added value of a therapy is high, despite not yet being clinically demonstrated. Clinical effectiveness can then be demonstrated by achieving outcomes that are agreed upon in a contract. The interviewees well received OBAs to collect more data, address the uncertainties, and most-importantly, spread risks between the government and manufacturers.

A well-considered agreement is essential. It is crucial to proactively determine the optimal time duration and payment adjustments, based on follow-up data, in line with the lifecycle of the drug and expected market competition because it is not exceptional that an alternative therapy will be found in a reasonable period.

*“You cannot change the rules during the game.”*

To get a view of which new drugs may enter the market, which affects the market dynamics, the government must conduct horizon scanning and take the pipelines of different companies into account at the time of negotiations of OBAs. For a hospital manager, market competition indicates renegotiating the absolute cost rather than the duration of the contract. According to the industry, after two or 3 years a re-analysis of the competitive market could be considered. Ideally, the cost of a drug is re-evaluated only for new patients since the cost for patients already receiving that therapy was previously agreed upon.

#### 3.3.1 Financing of agreements

To set up and implement OBSPs, the company and payer need to agree on the terms of the mechanism. The company initiates the proposal, outlining the details such as the payment amount and schedule, and outcome measures. Subsequently, the healthcare system (payer) assesses this proposal regarding its feasibility and engages in discussions to reach an agreement that is tailored to the healthcare system’s capabilities and legal boundaries.

*“Good insight into accounting data, taxation, and good knowledge of the stakeholders around the table, is needed before starting negotiations.”*

One interviewee proposed using a lump-sum financing model instead of a per-deed system to finance high-cost, curative therapies requiring long-term follow-up. In a per-deed system, reimbursement is based on individual interventions or services provided. With a lump-sum model, all costs associated with the therapy are covered under a single agreement. This means hospitals or physicians would receive a predetermined fixed payment for both treatment and follow-up, regardless of the complexity or the actual number of services provided.

#### 3.3.2 Payment initiation

Currently, reimbursement for ATMPs is dependent on completing the criteria outlined in Chapter IV of the Belgian list of reimbursed medicines (Box 1) (RIZIV, 2023a), with payments made when completing subsections at various times during follow-up. However, stakeholders noted that a better solution is needed.

Box 1 **Definition of chapter IV in the Belgian list of reimbursed medicines**

Chapter IV refers to the list of reimbursable pharmaceutical specialities that are subject to certain conditions imposed for medical and/or budgetary reasons. This means that the reimbursement is limited, for example, in terms of indications, target group, age, etc. In addition, prior authorization from the advising physician must be requested for these pharmaceutical specialities: this is an “*a priori*” check.

There seems to be some debate among stakeholders about who is responsible for initiating payment for ATMPs. Most stakeholder groups agree that it is the responsibility of the physician to decide if a patient meets the criteria, while some argue only one physician is not objective enough and that an external expert committee, i.e., the National Board of physicians or the orphan drug committee consisting of all sickness funds, relevant experts and the national payer, should be involved in the decision-making process because of the financial consequences. Others believe the physician can give important input by assessing and helping to determine whether the effect is sufficient, without making the decision.

Only a few stated that the manufacturer, the advising physicians of the sickness funds, the national payer or a supervisory body within the insurance of the national payer should be responsible for initiating the payment. However, one interviewee is a fierce opponent of having the payer decide on payment because they try to pay as little as possible. Appointing this responsibility to sickness funds was advised against because they lack the necessary data to make this kind of assessment. Sharing this data with sickness funds is not recommended as it could result in patient selection or varying reimbursement terms. On top of that, privacy issues may arise as they do not have the medical duty of confidentiality. A framework is needed to provide clear guidelines for payment initiation, i.e., the type of data that will be considered and what stopping rules will be used.

#### 3.3.3 Determination of outcome parameters and patient-reported outcome measures (PROMs)

Depending on the disease in question, it is important to have clearly defined, realistic, objective, and verifiable outcome parameters to measure clinically relevant health outcomes. Performance metrics that link payments to outcomes should be granular enough, i.e., more specific than overall survival, and easily measurable. However, accounting experts caution that the outcome parameters used may not be too easy to achieve because otherwise there is no conditionality, and it can be seen as trickery.

*“It must be credible, measurable, objective, relevant and verifiable because payers determine their funding and business income based on this.”*

Rational criteria must be conclusive, in line with company promises and agreed upon by all stakeholders, including patient organisations. However, the definition of effective medicine for the government differs from the one for patient representatives, and often registers are incomplete because important patient-reported outcome measures (PROMs) are not measured. Next to the merely clinical parameters, aspects of quality of life (QoL) and ease of use for the patient should also be considered according to patient representatives who expressed their concerns about how the government is holding on exclusively to scientific evidence for efficacy, rather than also considering these patient-reported outcomes as complementary evidence. Therefore, patients must be involved in data collection and most interviewees agree that PROMs are important to include as they will allow greater insight into qualitative and experience-related aspects. Currently, these QoL measures are often not included because the industry says that the committee for reimbursement of medicines (CRM) does not consider them as important as clinical outcomes, or they are seen as being too subjective. That is why they stick to scientific evidence for efficacy according to a patient representative.

Some even say that the government must demand fair and inclusive clinical trial designs that include PROMs. Whereas others referred to how PROMs make measuring outcomes more complex. For example, how does it feature in the agreement and schedule of payments? Will this be accepted as hard enough evidence for payers? A hospital manager pointed out that collecting QoL data is administratively burdensome and is more complex than getting the patient involved. To ensure the cost-effectiveness of PROM data collection, trade-offs must be made between the cost of collecting the data and its added value. To succeed in this, patient representatives and patient experts can have an advisory role in an early stage about the definition of performance, e.g., by measuring the QoL via PROMs.

Interestingly, one of the patient representatives is against using patient-reported outcomes mentioning they are too subjective. A few others join this and oppose the inclusion of PROMs, citing concerns that they are too vague, non-conclusive, subjective, and vulnerable to patient bias, as patients may be more susceptible to influence from manufacturers.

#### 3.3.4 Adaptation of payment based on effectiveness

Three positions are taken when talking about adapting the payment based on effectiveness. Almost all stakeholders agree that if the outcome is not reached, either the full amount must be refunded (binary all-or-nothing design) or the payment can be decreased (stepped design) if the drug is not as effective as promised after all. On the other hand, when the effectiveness surpasses expectations, augmenting the reimbursement basis up to a predefined threshold is seen as fair by several stakeholders. Nevertheless, there is doubt that authorities would be willing to endorse such price increments. Certain interviewees feel that a price increase is not justified as it is already accounted for in the value-based pricing. They believe that the benefit of a better-than-expected therapy should go towards the societal benefit, rather than augmenting the company’s profit.

Payment agreements for responders, non-responders and patients lost to follow-up should be made in advance. Several interviewees believed that when a patient is lost to follow-up, the payment stops. Who should be responsible for the loss of follow-up patients is an ethical issue. On one side, the company should not be penalised if a patient fails to attend a follow-up visit or when the hospital cannot keep in touch with its patients. Neither the hospital nor the physician should be held accountable for patients who drop out because it might cause discomfort or put stress on physicians.

*“It would not be fair for pharmaceutical companies to split the risk even when a hospital is not able to keep in touch with patients.”*

It is generally agreed that spread payment should stop when a patient passes away, even if the death is not related to the treatment. However, some argue that payments should continue regardless of the cause of death, while others believe that it depends on the specific circumstances surrounding the patient’s death. When a patient passes away due to an external factor some argue that the company should bear the risk, while the industry is not convinced that they should be held accountable in such situations. Two proposals are made. The first is to consider the average mortality rate during discussions, and the second is to pay the average cost for a patient who passed away and is thus lost to follow-up or can be considered as a non-responder.

#### 3.3.5 Population-level or individual-level adjustments

Choosing for payment adjustment on the patient or population level is a case-by-case exercise, dependent on differences in the contract and level of risk-sharing, the disease, the treatment characteristics, and the population size. A trade-off between the advantages and disadvantages (Table 1) must be made upfront. Most stakeholders, apart from the manufacturers, favour patient-level adjustment. There is agreement that effectiveness should be measured on an individual level, while adjustments are to be made on a subpopulation level. This level of standardisation makes sense because for the same effectiveness patients represent the same cost to society.

**TABLE 1:** Advantages and disadvantages of patient-level and population-level payment adjustment.

	Individual patient-level payment adjustment	Population-level payment adjustment
Advantages	- More detailed, finer adjustments	- For common diseases with a lower price per patient
	- Easier data analysis	- Mean response in real life compared to the mean response in clinical trials is the most pragmatic approach
	- Prevents the risk of no reimbursement for patients to whom therapy has potential ground-breaking effects because the overall population effect is too low	
Disadvantages	- Governance process is expected to be more complex	- Difficult to compare percentages in a small population with clinical trials, while at the same time generalizing too many risks to the lives of patients for whom the therapy could be ground-breaking
	- Individual value definition is more difficult	- Unfeasibility of the administrative burden for hospitals and hospital pharmacists in case of evaluation and payment once a year, as suggested because the national payer does not have the resources to make case-by-case invoices
	- Outcomes can differ individually	
	- Administrative feasibility (impossible to manage and administer to a large group of patients), balance between what is ideal and achievable	
	- Unpredictability when adjusting payments on an individual patient level in big patient populations	

### 3.4 Data collection

Gathering data is always challenging, which is why it is essential to define, in a formal contract, the type of data, how it will be collected and recorded, and the roles and responsibilities of all stakeholders involved, along with protocols for monitoring, managing, and processing data. Today much of the process depends on goodwill, without a framework in place.

A crucial factor in determining the success of annuities is the collection of good-quality data. Although, the industry has a well-developed pharmacovigilance system at a regulatory level for safety information, nothing remotely similar exists for outcomes. Ideally, to base payment terms on the outcome, it should be collected in a standardised and unambiguous way without being subjective or manipulable. There will be no one-size-fits-all approach, but rather a tailored one to each specific disease or case. Therefore, physicians believe that their input is crucial in discussions about the design of these agreements. In some cases, such as with Spinraza^®^ for treating spinal muscular atrophy (SMA), data collection has been insufficient to justify definitive reimbursement, although physicians have seen their patients’ progress.

*“With Spinraza* ^*®*^ *for SMA, measuring instruments cannot show much improvement in 2 years, but patients say they feel much better.”*

Interviewees proposed that to no longer have to depend on the goodwill of HCPs, manufacturers can make a lump sum available for collecting and processing data and publishing data by a data manager at the highest standards. This was backed by industry representatives who said that hospitals and HCPs are the ones that are burdened with data collection. Thus, they should be remunerated for their efforts because long-term follow-up must be organised at the hospital level. Others believe HCPs should not receive additional financing or extra incentives for providing reliable data. Moreover, to push discipline in centres for the long-term follow-up of patients and filing the data in the registers, they believe that data collection should be the basis for the funding of hospitals and HCPs. A similar practice is already used today, where part of the payment from the national payer to hospitals can be withheld to encourage hospitals to collect data and mitigate the risk of missing data. Payments could be linked to good practices of evidence-based protocols, e.g., appropriate remuneration (subsidy, budget, administrative fee) for patient follow-up efforts.

#### 3.4.1 Data collection sources

Four possibilities were proposed for data collection, the first possibility involves an *ad hoc* registry funded by the manufacturer, which is the most common type, but may not necessarily be aligned with other registries. The second option is a high-performance registration system with data extraction from the patient’s electronic health record (EHR) via healthdata. be. Whereas additional registries create an extra workload for hospitals, physicians, patients or authorities, HCPs understand that it takes accountability to fund expensive therapies and requires reliable data collected in an accurate and auditable way with trust from everyone. However, additional registers, next to the EHR, are frequently product-specific and require separate billing data to be sent to the national payer, resulting in an increased workload. To mitigate this, streamlining processes, eliminating duplication of work, and digitalising manual tasks could alleviate the administrative burden for HCPs.

*“There should be an incentive to look into what already exists, like eHealth and EMA databases, to recreate existing systems in a simple and pragmatic way, to not over-complexify data collection and create an additional burden.”*

The third option is to have, next to the EHR, a single platform for registering data, requesting reimbursement, and invoicing. Fourth and ideally, many referred to a completely automatic system that applies the only once principle and is system-2-system coupled. This means that data is entered only once, and the system is connected to other existing databases, i.e., the hospital database. In this way, eligible patients can be identified based on an algorithm that uses the coded data (EHR, lab parameters, etc.), and data can initiate or stop payment and notify when the patient has to be scheduled for a follow-up visit. In the interest of patients and care providers, registering data should be as easy as possible. Hence, most interviewees think the Belgian payer NIHDI must provide resources. Some also suggested that governmental grants could be considered to set up a digital, uniform, user-friendly system with electronic records for clinicians, which is compatible between hospitals so that data transfer is evident. The Belgian agency Sciensano must, in turn, provide the infrastructure to set up a uniform, integrated, user-friendly and standardized e-system with electronic records that are compatible between hospitals. For Sciensano, there is also a role to analyse these data and prepare reports for companies on request. For example, the analysis of the SMA Registry is done by Sciensano. Furthermore, for such an integrated system with strong data quality, the national payer should create and provide incentives for centres of excellence and integrate them into the recognition criteria of these centres. The first step is to set up pilots of such an integrated system with centres of excellence, which are recognized based on criteria determined by the national payer. It was emphasized by various stakeholders that a limited number of these centres of expertise such as hospitals and recognised treatment centres should be utilized.

#### 3.4.2 Data collection responsibilities

Currently, the responsibility for data collection often lies with manufacturers who set up and finance data collection infrastructure such as registries. To ensure that registers are well-filled and correctly recorded, these companies are required to inform each stakeholder about the importance of data collection and provide training and guidance to physicians on responsible prescribing, the importance of follow-up, and the type of patient data that should be collected.

*“Firms must think along about how they can help physicians to collect data; we must guide physicians and explain why it is necessary and communicate educationally. It is not because the database exists that it is filled in all at once.”*

According to the industry, authorities have plenty of unused information that the industry does not have access to or must pay for to gain access. Others claim that data of great value is often with manufacturers and not publicly available. Transparency was cited as a concern, as was the fact that registries disappear once they are no longer needed for HTA purposes. To prevent this, data collection should become a shared responsibility of HCPs, the national payer, hospitals and industry, and the commitments of all parties should be stipulated in a contract. By sharing the cost, the national payer and the manufacturers could gain control over the aspects of the process they want to govern. One proposal was that companies could facilitate registration, collection, processing, and analysis as they want to follow up on their health technology’s medical performance and see whether the predefined outcome has been reached. The government must improve the global tracking systems of patients, i.e., be more efficient and connected.

Patients play a vital role in measuring outcomes, and collecting, and registering data. However, there was no consensus on whether they could be expected to show up at follow-up consultations for the rest of their life with a one-off treatment. Patients need to be informed and be aware of their joint responsibility towards society and other patients, as well as what the implications are if they do not show up, e.g., payment stops if no evidence justifies public funding of the therapy. Thus, physicians and patient organisations were put forward to encourage the patient. To reduce the chance of loss to follow-up, it was proposed to oblige these responsibilities contractually and closely follow up on the patient. Interviewees had diverse opinions on whether (financial) consequences or sanctions should apply and what they should be when the patient is absent at follow-up consultations.

*“Who will pay if the patient does not show up? The firm or hospital, public health?”*

Some interviewees mentioned that it is unethical to hold critically ill patients accountable for the fact that the payments stop. They noted that the threshold to keep patients financially responsible would be high, given the pathology and vulnerable position of patients. Many others state that patients may be held accountable if they do not comply with reasonable conditions or commitments. Ideas such as a lower reimbursement rate and thus an increased co-payment for the patient were put forward as an incentive to strengthen engagement. Others propose a gentler approach with an alarming notification to patients that reimbursement is being put on hold.

#### 3.4.3 Ownership and access to the data

The interviewees held different views on who should own the data. Some suggested that it should belong to the patient, as they are the owner of their data, while others proposed the national payer, Sciensano, hospitals, centres of excellence, or industry.

Additionally, according to some, patients need total insight into their patient files and always know what is being collected about them better than currently with eHealth. Patients must be able to use applications that automatically update their patient files or registry, while hospitals must ensure the safety of the patient’s medical data.

Also, shared ownership or co-ownership was mentioned as it could contribute to scientific credibility and eventually lead to a better healthcare system, where real-world evidence is published in a register and where researchers can have *ad hoc* access to these registers and feed scientific research. However, there was consensus that whoever seeks to control the data, the data collection and analysis process should bear the associated costs.

According to a hospital pharmacist, there must be a standardised electronic dossier, next to the medical record, to which multiple parties can have access. Most important is that FAIR (findable accessible interoperable and reusable) principles are in place, and that access is granted based on the medical confidentiality obligation status. For example, in the best interest of patients, sickness funds can only have access to consumption data, and not to registries which contain medical information, because otherwise, a selection of patients or differences in reimbursement as well as privacy issues could occur based on the outcome data. Also, for manufacturers it is not clear if they should have access to certain patient data which could contain valuable information about their product. Even if the manufacturer supports the registration and processing of data, for privacy reasons manufacturers should only have access to the aggregated data as per sickness fund interviewees. To elevate concerns about privacy issues, granting third-party access to oversee the data collection is possible. For example, for the SMA Registry, there is a steering committee that governs data, and they assess the research questions before granting access to the often pseudonymised or aggregated data. Lastly, anyone with access to the registry should be able to perform analysis.

#### 3.4.4 Analysis and interpretation of data

A few respondents believe that the industry should not be involved in data collection or analysis and that it should remain neutral and separate from manufacturers. Some interviewees proposed that the analysis should be done by the payer or an independent governmental agency such as Sciensano. Next to this, an independent auditing body can be appointed, which verifies and determines if data was collected correctly. It can be agreed that within the government, certain experts, such as people from KCE, will supervise data analysis on patient records and other data and can author reports. However, such a control system with experts, who need insight into the patient record could pose some GDPR and privacy-related issues as patient data is strictly confidential and cannot be shared with the CRM workgroup or any manufacturer.

After a manufacturer submits its report to the national payer, experts in the working group at NIHDI should interpret, and analyse the outcome data to decide on continuing or renegotiating the agreement. However, the complex nature of highly specialised products such as ATMPs requires additional expertise beyond that of the internal evaluator at NIHDI. A pool of medical and other experts from organisations such as Sciensano, KCE, the Inter Mutual Agency (IMA-AIM), and the European Medicines Agency can be consulted to enable a scientifically objective evaluation process.

#### 3.4.5 International coordination of data collection

Almost all stakeholder groups agree on international coordination for data collection. For orphan drugs, cross-country data collection has already proven its added value to respond to remaining uncertainties and allow re-evaluation at a European level, rather than looking into small populations at the national level. Since more data can be pooled, the data and evidence are stronger, thus leading to more reliable statistical results. Another advantage is that sharing information internationally avoids duplicate efforts between countries. An example of this is the International Consortium for Health Outcomes Measurement (ICHOM) initiative, where experts agree on one dataset with parameters for measuring the efficacy of drugs across countries.

It is unsure whether data collected at a European or global level will be sufficient to base payment decisions on because countries are still interested in their own patient data for their national pricing agreements. Europe can approve something, but pricing and reimbursement will remain a national competence as member states want to keep jurisdiction over pricing and reimbursement nationally. Ideally, data on efficacy that is collected on a national level is put in one registry. There are already schemes based on joint criteria, such as Beneluxa, for joint HTA, negotiation and reimbursement. However, each country acts according to its insurance system. Consequently, one interviewee questions whether such international activity outweighs the misery of organizing such data collection in the short term. In an effort to generalise a register and increase usability, some countries are attempting to use the same standardised medical language, such as *SNOMED* ^1^.

*“There is an international register for Spinraza* ^*®*^ *. European Commission is putting a lot of effort into generalizing this, but progress is slow because of the complexity of speaking the standardized medical language like SNOMED for the system to use this.”*

Different Belgian parties were mentioned who could contribute to the joining efforts of data collection networks such as the Belgian cancer registry, Sciensano, the Federal Agency for Medicines and Health Products (FAGG), and the eHealth platform. Some stakeholders are more pragmatic: as it is already difficult on a national level, they propose to demonstrate locally how to succeed with national initiatives before going to a more global approach. Another interviewee clearly stated that Europe has a role in ensuring consistency across member states.

#### 3.4.6 Alignment of EMA and HTA data collection requirements

However, data submitted to EMA is collected to support granting marketing authorisation which is different from the data needed to inform HTA and reimbursement decision-making. However, alignment with EMA on data collection is seen as desirable by several stakeholders. EMA already uses registers in the context of the registration process, and it would be beneficial if data required in the context of financing and reimbursement could also be collected in these already available EMA registers.

In the long term, a European outcome collection mechanism should be possible and collaborative initiatives for clinical drug evaluation could be centralized. Next to the Beneluxa initiative, there is cooperation between the national payer and EMA. Back in 2019, there were already discussions between EMA and EUnetHTA about cross-licensing evidence generation and how to better collaborate on this. According to the interviewees, EMA is best positioned to push manufacturers in a European harmonised approach.

### 3.5 Governance

#### 3.5.1 Structural reform of the governance system

One suggestion that is put forward is to reform hospital financing in Belgium and prepare for the arrival of ATMPs with better horizon scanning and earlier consultation between manufacturers and the government. During the interviews, it was suggested that the government should build a platform for multi-stakeholder dialogue. This platform would aim to motivate stakeholders to share data, develop a common vision, and engage with each other to move towards a more value-based healthcare system.

*“We are at the end of the current system and new solutions have to be found by all stakeholders to create a win-win situation.”*

The interviewees hold differing views regarding the complexity of this reformed governance. One group believes that the complexity lies in the fact that there is a concern that part of the budget will be used to set up bureaucratic processes and procedures, which means that this money cannot be used to pay for treatments that directly impact citizens. Another group shares concerns about complicating governance due to the multi-year commitment, as payments in instalments involve greater administrative effort for hospitals than one-off payments. However, some interviewees find the governance reform to be simpler, as they see spread payments as a type of MEA with forms of *a posteriori* payments. They believe that committing to payments over several years can have positive budgetary effects if the processing and monitoring of spread payments are structured and digitized. Staff resources are especially important for managing OBAs because the patient has to be followed, registers have to be filled in, there is a need for horizon scanning, a solid monitoring process, a circuit of payments, re-examinations etc.

*“Hospitals find it difficult to administratively manage spread payments, their systems are not prepared for this. When they buy medicine, they are used to paying in one go. It is red tape.”*

One exemplified this administrative difficulty by saying that most therapies have co-payments from the patient. Thus, the patient should receive a bill, even if he does not have to pay anything. When paying in instalments, it is not possible to send an invoice for the same therapy several years in a row. However, it is feasible according to a hospital pharmacist if everything is planned well in advance with standing orders. According to a hospital manager, the MEAs lend themselves best to a flat fee convention-type system with patient follow-up by the hospital. Such a convention allows for some flexibility between the hospital and the government.

#### 3.5.2 External advisory board

Due to potential conflicts of interest between the company and the healthcare system, having a neutral third party with no direct financial interest would be beneficial. Many stakeholders support the establishment of an independent external advisory board to address conflicts of interest and oversee the performance-based agreements. While some call for an element of trust, many refer to the importance of independence and objectivity in data collection and analysis. The external advisory board would have a purely advisory (non-binding) role. Its responsibilities should not overlap with those of the CRM or that of the college of physicians, nor should it handle individual files.

This group of independent experts could include a neutral party, like a non-medical specialist. Other interviewees believe that sickness funds should not be part of the advisory board. Physicians and patient representatives should be part of it to play an evaluative role and have a say in future decision-making. However, one did not see the added value in involving patient representatives since it must be a scientific, objective debate and the patient’s interest is not the only one to be served.

One industry representative stated that such a body should not be involved in the negotiations of setting up the agreements but should manage registries and conduct structured analysis. This board would be responsible for determining performance parameters, separate from budgetary considerations, and would ensure adherence to the agreements. After 5 years, the board would review the overall system’s impact, independent of individual files, and make the necessary adjustments.

*“An external advisory board, which could be consulted, without obligation, before the deal is completed and that they share expertise with the company before submitting for reimbursement.”*

Instead of founding an additional body, some proposed that the Medical Evaluation and Monitoring Service (DGEC) at the NIHDI, which could investigate the data in registers, could take up this role.

#### 3.5.3 Transparency

It was acknowledged that the decision-making process and the following agreements may not always be transparent although they are funded by public money. Some stakeholders believe that there should be no secrecy about the type of agreement nor the terms and conditions of contracts and believe that, e.g., the used health outcomes and financing mechanisms, and all benefits and safety data should be made publicly available. In addition to contributing to scientific credibility, it is in the best interests of physicians, patients, the government, and corporations to be transparent to valorise data. Increasing the understanding of the risks, responsibilities, conditions and expectations related to these agreements would be a step forward.

Others stated that attention must be paid to ensure that increasing transparency on the financial level is not at the expense of accessibility of medicines in Belgium as there is legitimate concern around competition policy and trade secrets. Hence, the transparency exception for the financial elements of agreements. To increase insight into the financial side of the contracts, a multi-stakeholder governance system is needed with appropriate safeguards that the information that is made transparent cannot be misused, e.g., for commercial purposes. The first steps are set with a Belgian act that will increase transparency by imposing parliamentary insight into MEAs (Kamer, 2020).

### 3.6 Regulatory compliance with both European and national accounting rules

The Belgian health system works with a fixed budget per year, and annual budget cycles, meaning that the full cost of a service/treatment delivered in a specific year must be accounted for in the budget of the FPS Public Health and the NIHDI in that same year. The payer’s budget is thus not structured to recognize health interventions whose value accrues over years or even decades. Next, for hospitals to spread an invoice over more than 2 years can be challenging. Consequently, multiannual invoicing may constitute a budgeting barrier for both the payer and the manufacturer, who are used to operating with annual budget cycles.

Currently, the European System of Accounts (ESA) dictates to the member states how they should report and budget their health expenditures to the EU. Eurostat is the controlling body and can declare a government at fault if it does not follow the rules. ESA was described as a static, yet complex registration system that everyone has to use although it has no legal authority. The way a new payment system is worked out must be checked with ESA to be compliant or a procedure should be initiated to ask Eurostat for an exemption. According to accounting experts, annuities are not possible because write-offs are not provided in ESA. As mentioned by several, the biggest problem is that for according to Eurostat everything should be billed in 1 year.

Across the interviewees, opinions differ on whether structural adjustments are needed to accommodate specific payment types via legislative amendments. The interviewees suggest that there needs to be a discussion between the national payer and European bodies to ensure that the local and European legislations are aligned. They also suggest relaxing the accounting rules on a European level, because they were not developed with ATMPs in mind.

### 3.7 Data protection and privacy

Three main data protection themes were raised by the interviews.

#### 3.7.1 General GDPR issues

The GDPR is one of the most stringent regulations for personal data privacy. As spread payment requires the collection of personal health data, regulations to safeguard these data, following the GDPR, will make the execution of such an agreement more complicated and potentially more expensive. What type of data can be legally made available, to whom, and for what purpose should be discussed? Also, safeguards should be in place to prevent any misuse of data, e.g., suboptimal analyses without peer review.

#### 3.7.2 Privacy of the patient

When it comes to sharing data, privacy issues arise. Precaution is needed to ensure confidentiality, e.g., personal data should not be linked to outcome data in the same database. Manufacturers, nor the government need to get access to personal patient-related data. While some argue that manufacturers are better equipped with cross-country data to perform analysis related to their products, concerns about the anonymity of patient data were raised during the interviews.

*“Pharma should not know to whom the product is supplied.”*

Society is committed and wants to learn; this is a justifiable reason to ask the patient to commit and accept that data is shared. The more firewalls between industry and institutions, the better the protection of the patient. However, this is not always as easy. In the case of rare diseases, the privacy commission will have to decide what results can be shared to protect personal data, because there is only a handful of patients in one country. Therefore, one interviewee proposed to let it happen on an international level.

#### 3.7.3 Consent of the patient

Although ATMPs and potentially spread payments covering these will require life-long follow-up of sensitive personal data. Today, making data transparent is no problem; e.g., the medical eHealth file can already be shared with another physician. However, one interviewee believes that the patient will have to give extra informed consent to collect his/her sensitive personal data and share it, in an aggregated or anonymous fashion, with other stakeholders such as the national payer and manufacturers for payment models. Next, a patient can withdraw his consent at any time, which may have implications for the registry and add complexity to the payment model.

## 4 Discussion

ATMPs represent a promising frontier in medicine, offering potentially life-changing treatments for a variety of diseases. However, they also come with significant financial implications. Currently, the cost of these therapies remains uncertain from the government’s perspective, while companies encounter difficulty in accurately calculating their expenses. Compounding this challenge is the fact that ATMPs are frequently developed for rare diseases, resulting in small patient populations. To offset development costs and ensure profitability, these therapies often come to market at high prices.

While existing literature extensively covers the determination of these costs and pricing strategies, this study focuses on how such therapies, despite their high price tags, can reach the patient through implementing an OBSP system. This requires resolution on opinionated positions related to the discussed topics such as the payment structure, data collection, determination of outcome parameters, governance, and privacy and transparency concerns.

Table 2 compiles the necessary conditions that, fulfilled together, are seen to be sufficient for the successful implementation of OBSP (Pangarso et al., 2022).

**TABLE 2:** Key conditions for OBSP implementation: Addressing agreement and Refinement needs across payment structure, spread payments, outcome-based agreements, data collection, governance, privacy, and transparency.

Payment structure
Agreement needed on:
⁃ The MEA structure for ATMPs
⁃ The organization of payment flow

Spread payments and outcome-based agreements
Agreement needed on:
⁃ The duration of the agreement
⁃ Taking market competition into account
⁃ Payment trigger
⁃ The determination of outcome parameters
⁃ Reimbursement level linked to the chosen outcome measures

Data collection
Need for:
⁃ Robust data collection infrastructure
⁃ Financing of infrastructure and data collection process
⁃ Solutions for follow-up of patients moving abroad

Governance
⁃ Refinement needed of the roles and responsibilities (cfr. Conflict of interests)
⁃ Proposition of an external governance body

Privacy and transparency
Transparency needed on:
⁃ The price and/or price mechanism
⁃ The type of data to address uncertainties
⁃ The type of agreement
⁃ Data protection legislation (i.e., GDPR) and national implementing rules

### 4.1 Payment structure

Without a consensus on what constitutes fair pricing, the implementation of new payment models is anticipated to encounter substantial challenges. Interviewees highlighted that authorities often lack precise information regarding the true cost of therapies, while manufacturers advocate for value-based pricing. This disparity raises pertinent concerns regarding the concept of fair pricing. Stakeholders have proposed a solution involving upfront compensation coupled with post-HTA adjustments to ensure fair pricing. Additionally, interviewees emphasized the necessity for reforming the current financing flow. However, before proceeding with such adaptations, a comprehensive understanding of the broader system, and careful consideration of long-term implications are deemed crucial (RIZIV, 2023a).

### 4.2 Spread payments and outcome-based agreements

Spread payments may be necessary to accommodate the budgetary impact challenges (pharma, 2022) and may provide a solution to the clinical uncertainties of ATMPs (Maes et al., 2019). However, it is insufficient to merely spread payments as it may limit future healthcare policy initiatives. It is necessary to consider immediate and long-term implications on research and development, pricing strategies, patient access, and overall sustainability of the healthcare system. Therefore, combining spread payment with an outcome-based mechanism allows for price modelling based on efficacy data from follow-up, literature, and real-world evidence generation.

#### 4.2.1 Determining outcome parameters

Reaching agreement on the disease-related outcome parameter (e.g., population, and survival rate) and the frequency with which it is measured the economic factors (e.g., competition, opportunity cost, and inflation) is crucial to mitigating and sharing risks between the payer and the manufacturer. Besides the purely clinical parameters, QoL measures and PROMs were proposed as relevant inputs to link payments to outcomes if they are clearly defined, realistic, objective, and verifiable. Therefore, it is necessary to allow the affected patients the opportunity to express what QoL means to them (Ulrich, 1987).

#### 4.2.2 Data collection

The interviews covered four data collection possibilities, with a preference for an automated system-to-system coupled platform, adhering to the “only once” principle. Implementing FAIR principles is crucial for achieving a data-driven healthcare system that ensures uniform, transparent, and secure access to healthcare information. Maintaining a robust, flexible, and adjustable data collection system is vital to minimize burdens on patients and physicians (Rey et al., 2022). Efforts towards these goals are underway by establishing the Belgian Health Data Agency (HDA) and developing a uniform, integrated, user-friendly, and standardized e-system (Vandenbroucke, 2023; Belgische Kamer van volksvertegenwoordigers, 2022; Belga, 2023). Also, HCPs and patients have a significant role in robust data collection. It is therefore essential to establish consensus among stakeholders on incentivizing their contributions.

In line with initiatives such as EHDEN, TEHDAS, and DARWIN (EHDEN, 2022; TEHDAS, 2022; European Medicines Agency EMA, 2023), aligning data collection for pricing and reimbursement with EMA’s post-launch data requirements is considered a viable option for harmonizing and centralizing clinical drug evaluation. Our interviewees and previous research findings endorse this perspective (Rey et al., 2022). Moreover, this approach is consistent with efforts like the RWE platform outlined in the Belgian national payer’s roadmap for reforming reimbursement procedures (RIZIV, 2023b).

### 4.3 Governance

Defining outcomes, generating evidence, handling the administrative burden, and ensuring compliance with data protection regulations constitute barriers to implementing outcome-based reimbursement models (Michelsen et al., 2020; Callenbach et al., 2022). Many interviewees therefore advocate for establishing an independent multi-stakeholder advisory board, either at the European level, aligned with the HTA regulation (The European Parliament and The Council of the European Union, 2021), or at the national level, considering final pricing and reimbursement decisions fall within regional or national competence. A multi-stakeholder governance approach, supervised by a public entity independent of economic interests, is essential for effective data management. International cooperation, exemplified by European reference networks, is deemed advantageous for consolidating data, avoiding duplication, and promoting interoperability.

Led by an academic research centre, this board would include various stakeholders such as HCPs, patients, policymakers, and industry representatives. Recognizing the divergent views, values or interests among stakeholders, the board’s primary role would be to provide a platform where all parties could voice their concerns, e.g., related to antitrust competition policy and trade secrets. Such early-on consultation between stakeholders is crucial for promoting understanding, and collaborative decision-making processes.

### 4.4 Privacy and transparency

Increasing transparency is essential for enhancing scientific credibility and maintaining trust among stakeholders. One suggestion to promote transparency that emerged from the interviews which is also supported by research conducted in the Netherlands (Callenbach et al., 2022) is to foster a transparent decision-making process and make the terms and conditions of final agreements publicly available. However, it is crucial to ensure that efforts to improve financial transparency do not compromise the accessibility of medicines (S and M, 2024; E et al., 2022). Furthermore, safeguarding patient and user trust requires prioritizing data protection throughout the data lifecycle, including collection, storage, and utilization. The GDPR is a vital framework for ensuring this data security and trust. Another effective strategy for building trust is to provide patients with access to a dashboard where they can review their data and insights, as demonstrated by previous research (Rey et al., 2022). By implementing these necessary measures, healthcare systems can work towards enhancing transparency, while protecting privacy, and fostering trust among all stakeholders.

### 4.5 Strengths and limitations of this study

A few limitations of this study must be considered. Different stakeholders may have interpreted and responded to the same questions differently based on their backgrounds, personal biases, interests or agendas and experiences leading to a variability in responses which complicated extracting the most salient themes or insights from the interviews. Due to the nature of the participants, the survey is likely biased in the positive direction, potentially impacting the representativeness of findings and generalizability of the results to a broader population. Generalizability of the results might be limited to countries with a social security healthcare system, similar to that of Belgium, which may limit the applicability of the findings to other countries or regions with different healthcare structures and reimbursement policies.

### 4.6 Further studies

Future research should focus on the ethical, financial, and other boundaries that should be considered to put a framework in place that refines the proposed necessary and sufficient solution elements to overcome the barriers to implementation. These barriers tend to be influenced by numerous interrelated factors and thus require a deeper understanding of the underlying dynamics and interactions. The authors suggest putting the qualitative results in perspective by employing the method of boundary critique from critical system heuristics (Ulrich, 1987; Ulrich, 2012), facilitating critical solutions that align with the common understanding of all involved and affected stakeholders. To strengthen the validity and reliability of future research endeavours, organizing focus groups would be valuable for elaborating on the findings and generalizing them to a broader population.

## 5 Conclusion

Our research contributes to the theoretical and practical knowledge of implementing an OBSP model for ATMPs by discussing necessary and sufficient conditions. To increase the use of OBSPs across Europe, payers, manufacturers, HCPs and patients need to embrace the shift towards data-driven payments for high-cost, curative therapies and agree on the generation of evidence and determination of outcome parameters, management of the administrative burden, data protection, (data platform) governance, and privacy and transparency concerns. Adapting the payment system would streamline the process and increase transparency next to facilitating the implementation of innovative payment models.

## 6 Nomenclature

### 6.1 Resource Identification Initiative

To take part in the Resource Identification Initiative, please use the corresponding catalogue number and RRID in your current manuscript. For more information about the project and for steps on how to search for an RRID, please click here.

## Data Availability

The datasets presented in this article are not readily available because the transcripts, although pseudonymized cannot be shared to safeguard the integrity of the interviewees. Requests to access the datasets should be directed to contact.isabellehuys@kuleuven.be.

## Footnotes

1 SNOMED CT (systematized nomenclature of medicine clinical terms) is a medical standard for documenting and coding medical data. SNOMED CT consists of a collection of medical terms coded to be processed by a computer. It includes areas such as illnesses, symptoms, procedures, treatments, devices, and pharmaceuticals.
