# Supplementary material for: Towards implementing new payment models for the reimbursement of high-cost, curative therapies in Europe: insights from semi-structured interviews
Source: Front Pharmacol. 2025 Jan 20;15:1397531. doi: 10.3389/fphar.2024.1397531 (PMC11788164; doi:10.3389/fphar.2024.1397531)
Supplement: Supplementary file 1 [file DataSheet1.pdf]

Sample size

Interviews will be conducted until data saturation is reached, meaning no new insights are being found. When a researcher observes similar responses repeatedly, the researcher becomes empirically confident that a certain topic is saturated (1). The desired number of interviews is approximately 30, with a diverse sample and every stakeholder group represented:

- Physicians: 3 individual physicians, from different therapeutic domains
- Pharmacists: 2 hospital pharmacists
- Hospital managers: 3 from different hospitals across Belgium
- Domain-specific experts: 2 legislative experts and 2 accounting experts
- Industry (organisations): 2 representatives of industry associations (Pharma.be and EFPIA), as well as 3 representatives of pharmaceutical companies
- Patient organisations: 4 representatives of different organisations, from different therapeutic areas
- Policy makers: 3 representatives of NIHDI, 1 of KCE, 2 of the Federal Public Service and 1 of healthdata.be
- Belgian Health Insurance Organisations: 3 representatives of different health insurance organisations

We aim to include six to ten participants in the focus group discussion consistent with recommendations formulated in the AMEE Guide No. 91. (2), each participant being a representative of one of the stakeholder groups mentioned above. The ideal composition of the focus group requires the presence of: a physician, a pharmacist, a hospital manager, an industry representative, an NIHDI representative, a Federal Public Service representative, a KCE representative, a Belgian health insurance organisation representative, a legislative expert and a public accounting expert.

1. Kerr C, Nixon A, Wild D. Assessing and demonstrating data saturation in qualitative inquiry supporting patient-reported outcomes research. Expert Review of Pharmacoeconomics & Outcomes Research. 2010;10(3):269-81.
2. Stalmeijer RE, McNaughton N, Van Mook WN. Using focus groups in medical education research: AMEE Guide No. 91. Med Teach. 2014;36(11):923-39.
3. Palinkas LA, Horwitz SM, Green CA, Wisdom JP, Duan N, Hoagwood K. Purposeful Sampling for Qualitative Data Collection and Analysis in Mixed Method Implementation Research. Adm Policy Ment Health. 2015;42(5):533-44.
